# Burden of Mortality and Morbidity Caused by Snakebites Contributes to Economic Loss in a Rural Population in India

**DOI:** 10.3390/toxins18060250

**Published:** 2026-05-29

**Authors:** Swapnil Kiran, Siripuram Srinivas, Karthikeyan Vasudevan

**Affiliations:** 1 CSIR Centre for Cellular and Molecular Biology, Hyderabad 500007, India; swapniliiserb@gmail.com (S.K.); srinuuohyd@gmail.com (S.S.); 2 Academy of Scientific and Innovative Research (AcSIR), New Delhi 201002, India

**Keywords:** economic cost, human–animal conflict, public health, snakebites, snowball sampling, socio-economic impact

## Abstract

Snakebite envenoming is a major public health concern in India that causes economic hardship for the rural populations. We estimated the per capita economic burden of snakebites in a rural population by quantifying mortality and morbidity rates. We interviewed for outcomes of snake envenomation of 541 participants from 205 villages in Jagtial, Telangana, from 2010 to 2020 using a community-based snowball sampling approach. Snakebites caused 24.21% morbidity and 12.75% mortality. The age-adjusted mortality rate and age-adjusted morbidity rate were 11.72 and 22.8 per 100,000 people, respectively. The overall annual burden of snakebites was 31.96 Disability-Adjusted Life Years (DALYs) per 100,000 people. The mean annual earning opportunity cost and the mean annual mortality cost were USD 321.27 and USD 24,016.54 per person, respectively. We highlight the need for targeted public health interventions such as monetary compensation and community support schemes to reduce the morbidity and mortality rates in rural areas.

## 1. Introduction

Snakebite poses a serious threat to rural communities in Asia, Africa, and Latin America [1,2,3,4,5,6,7]. Several groups of investigators have sought to determine the relative risks, mortality and morbidity of snakebites and their sequelae (e.g., Gutiérrez et al., 2017; Menon et al., 2025; Suraweera et al., 2020; World Health Organization [1,7,8,9]), but published figures are often based on rough estimates and speculations. The global strategy is to halve the number of snakebite envenomations and deaths by 2030 [10,11,12]. India particularly has a large burden, with over 58,000 deaths every year and only 20–30% of the victims seeking treatment [1,13].

The triage of snake-human interactions involves: the abundance of venomous snakes, the treatment for snakebites, the social milieu, the economic condition, and the level of awareness about snakebite treatment [14]. The impacts of snakebites are disproportionately large on rural communities [2,14,15,16,17,18] and the mortality rate is closely linked to per capita GDP (Gross Domestic Product) and human development index [19]. Therefore, it is essential to quantify the economic impact of the ‘big four’ species (Common krait (*Bungarus caeruleus*), Indian cobra (*Naja naja*), Russell’s viper (*Daboia russelii*), and saw-scaled viper (*Echis carinatus*) [20] as well as other medically relevant snakes [21,22,23,24] responsible for snakebite envenoming in India [2,25].

Snakebites result in full recovery, death, or disability/morbidity. Disability-adjusted life years (DALY) is a standard metric used to estimate the burden of disease, wherein one year of healthy life lost is equivalent to one DALY [2,19,26]. The total DALYs for all age groups and genders represent the gap between a perfectly healthy population that is free from illness and disabilities and one that is exposed to snakebites [2]. Estimates of morbidity, mortality rate and the financial burden on victims inform the policy makers about allocation of resources for treatment [16]. Although efforts have been made to quantify the global economic burden of snakebites, comprehensive data on morbidity and national-level cost estimates for India are still lacking [27]. Data pertaining to treatment outcomes and the economic burden of snakebites are difficult to collect, as victims are reluctant to share information [17]. Victims of snakebites face social stigma and do not readily reveal their experience [17,28]. Populations at risk of snakebites are often spatially isolated and victims often do not reveal their snakebite experience as it is culturally associated with taboos. Therefore, capturing the incidence of snakebites efficiently requires a process of tapping the knowledge of the community members about victims through referrals. This would lead to a network of referrals in the population and include those who might not be effectively captured in a random sample and reduce survey costs. The spatial heterogeneity in snakebite incidence might also exclude a high concentration of victims from the random sampling approach. To overcome these issues, we used a community-based snowball sampling approach with some modifications. It uses existing community knowledge and networks, leading to referrals of victims to document those that remained undocumented in the population. Using this approach, we quantified the economic burden of snakebites in the rural population of a district, since it is the smallest administrative unit for public health management in India. We conducted the study in Jagtial District in southern India and quantified mortality, morbidity rates, and per capita economic burden of snakebites. The findings highlight the high economic costs of snakebites, including the morbidity costs, so far undocumented, and the need for revisiting the different approaches used to assess this burden. This has implications for strengthening rural healthcare, financial risk protection, and awareness.

## 2. Results

### 2.1. Incidence and Outcomes of Snakebite

Using the community-based snowball sampling approach, we interviewed 541 participants (Table 1) from 205 villages in Jagtial District who were victims or their kin and had experienced snakebite between 2010 and 2020 in the study area. The district hospital data revealed a cumulative incidence of 104.42 (CI: 98.14, 110.99) per 100,000 people from 2015 to 2020, with a crude mortality rate of 1.01 (CI: 0.49, 1.87) per 100,000 people. Whereas, the cumulative incidence for the same duration from our community survey was 55.79 (CI: 50.15, 61.89) per 100,000 people with a crude mortality rate of 5.94 (CI: 4.20, 8.15) per 100,000 people.

### 2.2. Demographic Profile of Snakebite Victims

There was a significant difference in the proportion of victims belonging to different age groups (Pearson’s χ^2^ = 150.74, *p*-value < 0.05). Agriculture workers made up 46.40% (n = 251) of the victims, which constituted the largest group under occupation. People employed in other sectors, such as government, medical professionals, teachers, village administrators, and business, together constituted 35.30% (n = 191) of snakebite victims. Victims in the age group 15–60 years and unemployed constituted 9.80% (n = 53) of the respondents. Victims who were <15 years, >60 years and students >15 years of age and not part of employment constituted 8.50% (n = 46). Inhabitants of traditional housing were more prone to snakebites (65.06%, n = 352) than those who inhabited concrete housing (32.90%, n = 178). The remaining victims (2.03%, n = 11) did not disclose the type of house they lived in. Among the participants, 4.44% (n = 24) of the participants belonging to the age group 0 to 14 and 7.39% > 60 years (n = 40) were the least exposed to snakebites among different age groups. The remainder of the population—adults (15–59 years) were the most impacted by the snakebite (88.17%, n = 477).

### 2.3. Circumstances of Snakebite Incidents

A majority of the victims (38.45%, n = 208) or those who witnessed the incident were unable to identify the snake that inflicted the snakebite. Among those who identified the snakes, the following venomous snakes were involved: *Naja naja* (46.25%, n = 154) and *Daboia russelli* (39.34%, n = 131), *Bungarus caeruleus* (12.61%, n = 42) and *Echis carinatus* (0.90%, n = 3). Few victims reported snakebites caused by non-venomous snakes (see Supplementary Figure S1A). A majority of the victims (64.33%, n = 348) were bitten in the foot (see Supplementary Figure S1B). Snakebites occurred predominantly in the agricultural fields (55.08%, n = 298). Some of them (32.16%, n = 174) were bitten in or around their house (see Supplementary Figure S1C). Many snakebites (42.51%, n = 230) occurred during the day between 1200 and 1900 h. A smaller number of snakebites occurred the morning before 12 noon (33.83%, n = 183) (Supplementary Figure S1D). The timing of the incident was associated with the species of snake involved in the incident (Pearson’s χ^2^ = 33.61, *p*-value < 0.05) (see Supplementary Table S1.1).

### 2.4. Snakebite Burden

Among the 541 victims, 62.29% (n = 337) recovered completely, and 24.21% (n = 131) suffered morbidity. Among the victims, 12.75% (n = 69) died, and 0.74% (n = 4) of the victims chose not to respond about their condition. A sizeable proportion chose to access treatment in a hospital (78.18%, n = 423). Among them, 60.52% (n = 256) recovered completely with no morbidity associated with the snakebite. Among the victims who accessed treatment, 27.66% (n = 117) suffered morbidity, and 11.82% (n = 50) died due to complications. Notably, 30.81% (n = 130) of them visited more than one hospital to access treatment after the incident. Some of them (0.95%, n = 4) visited four different hospitals to seek treatment. Treatment cost was significantly different in the time intervals for the victims to reach the hospital (Kruskal–Wallis χ^2^ = 8.25, *p* < 0.05). Treatment costs for the victims who accessed treatment within one hour of the incident and between two and five hours were significantly different (Wilcoxon rank sum test, *p* < 0.05; Supplementary Table S1.2). There was no difference in treatment cost with the age, the gender of the victims, or the species of the snake involved in the snakebite.

The proportion of age-wise victims seeking treatment did not vary significantly. A greater proportion of males (82.48%, n = 259) sought treatment than females (72.25%, n = 164) in the population. The age-adjusted mortality rate was 11.72 per 100,000 people (95% CI: 8.08, 13.20), which corresponds to an average of 1.07 per 100,000 people per year (95% CI: 0.73, 1.20). The age-adjusted morbidity rate was 22.8 per 100,000 people (95% CI: 17.23, 28.33), which translates to about 2.07 per 100,000 people per year (95% CI: 1.57, 2.57) (Table 2 and Table 3).

The overall burden of disease quantified as disability-adjusted life years (DALY) in the district for the study period was 3464.12 (CI: 3462.92, 3465.32), and it translated to 31.96 (CI: 31.95, 31.97) DALY per 100,000 people annually. Cumulative healthy years lost due to disability (YLD) was 771.66 (CI: 771.43, 771.89), and it was 7.118 (CI: 7.116, 7.121) YLD per 100,000 people annually. Cumulative years lost due to premature death (YLL) were 2692.46 (CI: 2688.29, 2696.63), and it was 24.84 (CI: 24.80, 24.88) YLL per 100,000 people annually in the district. The DALY for females and males were 1585.45 (CI: 1583.44, 1587.46) and 1878.67 (CI: 1877.19, 1880.16), respectively (Table 4). This amounted to 28.75 (CI: 28.71, 28.79) and 35.28 (CI: 35.25, 35.31) per 100,000 people annually for females and males, respectively.

The overall DALY was 1.22 times higher in males than in females. The YLD for the ‘recovered without morbidity’ group of victims was smaller than that for the victims who suffered ‘long-term morbidity’. The mean annual per-victim cost of treatment for snakebites was USD 639.15 (CI: 606.10, 672.21). The mean annual cost incurred per victim for loss of earning opportunity during the study period was USD 321.27 (CI: 307.12, 335.42). The mean annual cost incurred per victim for mortality was USD 24,016.54 (CI: 19,120.71, 28,912.36; see Supplementary Table S1.3).

## 3. Discussion

### 3.1. Incidence and Outcome of Snakebite

Snake envenomation has gained importance as a public health issue due to the large burden of deaths [1,2]. In South Asia, one of the major difficulties associated with the prevention and control of snakebite envenoming is the paucity of data on mortality [29]. Access to such data is fundamental to planning and implementing programs that could reduce deaths and trauma caused by snakebites [30,31]. Our data shows significant differences between the hospital-based records and the community-based reports, and it offers evidence for accurate measures of snakebite envenomation at the community level in a District. In the district, the cumulative incidence of snakebites from 2015 to 2020, based on hospital records, was higher than that captured in the survey. However, mortality in the hospital data was lower than that documented by the survey. This difference might be due to the cases referred from nearby districts accessing treatment from the district hospital at Jagtial due to proximity or preference. Mortality caused by snakebites is clearly underrepresented in the public health records due to the taboos associated with reporting them [9]. Since it is important to accurately estimate the mortality caused by snakebites, standardized approaches are required. However, taboos associated with snakebites might prevent reporting and their enumeration. An approach that incentivizes self-reporting by victims might reveal the accurate burden of snakebites. We used a snowball sampling strategy to obtain referrals by village heads, ASHA workers, the forest staff, and victims to maximize the capture of snakebite incidents. In a typical village setting, referrals from victims or community leaders built trust and also encouraged participation of victims who might not have otherwise shared information. Community-based surveys captured burden more effectively than public health system-driven surveys that relied on passive reporting (see Table 5). Although it captured mortalities and morbidities in excess of those captured in the hospital records and the outcomes of treatment, it underrepresented victims who recovered fully.

The age-adjusted mortality rate (1.07 per 100,000 people) in Jagtial was three-fold more than the current estimates at 0.33 per 100,000 at the country level [9]. However, the MDS estimated age-adjusted snakebite mortality rate of around 4.5 per 100,000 population [1,13] could be an overestimate. This discrepancy could be attributed to differences in the methodologies used. Therefore, necessary modifications to the sampling approach could lead to accurate estimates of the burden of snakebites. This, in turn, would help in identifying vulnerable populations and deliver measures to reduce the impacts on them. After standardization of this approach, it could be scaled up to the country level to address the WHO’s goal of halving snakebite death and morbidity rates by 2030.

### 3.2. Demographic Profile of Victims

The age group between 0 and 6 years and 45 and 64 years experienced high mortality, and the age group between 40 and 64 years experienced high morbidity. This highlights the number of lives and productive years lost due to snakebites, underscoring the need for targeted intervention. It is critical to further evaluate the specific circumstances that might have led to snakebites among children and elderly people in the population. The impact of snakebites is disproportionately high on the working population [1,9,16,31,32,33,34,35], as a majority of victims were adult and middle-aged (15–59 years) farm workers. Adult males faced a high risk due to prolonged exposure during farm-related activities. In Brazil [36] and India [9,13], the increase in the number of victims who are adult males has been attributed to the proportion of fewer women employed in farm work. Due to behavioral differences, middle-aged adults, males, field workers, and individuals with a poor level of education face a high risk of snakebites [37,38,39]. Exposure to snakebites increased through manual sorting with bare hands, working with bare feet, pruning, harvesting, or irrigation activities. Although we could not collect this data, the activity of the victims at the time of the bite suggests a possible association. The snakebites were inflicted on the feet or on the legs of victims [40,41,42]. In Brazil and Sri Lanka, snakebite victims were associated with farming activities [36,43]. Among the victims who were not farm workers, envenomation predominantly occurred in and around their houses. These incidents were associated with inadequate protection at traditional houses (for definition, see Supplementary S2.1). When suitable microhabitats and food are available, snakes seek shelter and forage around human habitations. The increased proximity to humans and venomous snakes further increases the risk of snakebites [44].

### 3.3. Circumstances of Snakebite Incidents

*Naja naja,* followed by *D. russelli,* were responsible for the high proportion of snakebites and they also cause a majority of snakebites in India [45,46,47]. From our data, *N. naja* and *D. russelli* were involved in bites during the day, whereas *B. caeruleus and E. carinatus* were involved in bites during the night or early morning hours, respectively (Figure S1E). Few studies have correlated the activity of these snakes with the pattern of snakebites caused by these species of snake [43,48]. In this study a majority of the victims could not identify the snake species at the time of the bite (Figure S1A), which highlights a critical challenge for effective snakebite management [47,49,50].

### 3.4. Snakebite Burden

A large proportion of snakebite victims in the study survived, indicating that dry bites might have played a role, and global datasets suggest that they constitute 50% of bites [51]. Our study revealed that roughly one in four victims suffered from morbidities. To our knowledge, this is the first comprehensive assessment of the morbidity and the economic burden attributable to snakebites in a population from India (Table 5). While most snakebite studies report incidence and mortality due to snakebite [1,5], not much attention is paid to the population of victims who survive but suffer morbidities for the remainder of their lives, or die many years later due to complications (Figure S1E). The high level of morbidity might be due to the delay in receiving appropriate treatment (Figure S1F), probably due to lack of awareness about snakebites, or due to ineffective treatment. The mortality rate is generally low with increasing level of access to healthcare and sufficient antivenom. Delay in accessing healthcare facilities was due to the distance to the hospital, lack of transportation, and dependence on traditional healers. Limited knowledge of treatment could prolong envenoming effects, leading to morbidity and even delayed mortality [52,53,54]. Emergency ambulance cover and awareness campaigns could improve rapid access to treatment in a rural setting [55,56,57].

The common reason for delays in accessing treatment was the behavior of victims to seek treatment from faith healers instead of accessing the hospitals. This points to the influence that faith healers have on the outcomes of snakebite treatment. Because the faith healers can be an important source of information on snakebites in the rural areas [58], we suggest that the public health system should: (i) involve them in recording and reporting of snakebite incidents, (ii) encourage them to support victims to access treatment in the nearest district hospital.

Snakebite survivors reported fear of bites and insomnia, and that it impacted their daily activities. Snakebites can cause a variety of mental health issues such as depression, post-traumatic stress disorder, and anxiety in victims, which might influence the victim’s well-being [59]. The morbidity cost estimated in this study did not account for these costs. Therefore, the economic burden of morbidity could be an underestimate of the actual cost incurred by the snakebite victims.

A large number of households (N = 193) expressed financial strain due to out-of-pocket expenses for the treatment of the snakebite, with the majority (80.82%) seeking treatment in private hospitals. Although the government-run hospitals provide free treatment for snakebites, victims sought treatment for snakebites at private hospitals, incurring high costs [60]. The cost of snakebite treatment in India is affected by a variety of factors: healthcare-seeking behaviors, traditional beliefs, and the accessibility of medical attention. Community education and improved antivenom distribution, particularly in areas with the highest prevalence of snakebites, can reduce snakebite deaths [13]. This might discourage victims to seek treatment in hospitals. Although previous studies indicate that factors such as age, gender, and snake species significantly influence the cost of treatment [61], we did not find such a relationship. The absence of key clinical variables—such as envenoming severity, length of hospital stays, antivenom dosage, and pre-existing comorbidities might have influenced the calculation of cost. It underscores the need for patient-level clinical data, precise hospitalization costs, and the use of formal health-economic methods for snakebites.

The DALY for snakebites in this study was lower than that estimated for Nepal [30], but higher than that estimated in Sri Lanka [19], but direct comparisons are limited by differences in scale, methods, and disability weights. The DALY estimates can vary across studies depending on the population structure and the model used. The estimate for healthy years lost for the age group below 6 years was the highest across all the age groups for the district. The burden of the snakebite was similar in different age groups except for the age group > 65 years, where there was a decline in the DALY. Few reports substantiate the burden of snakebites among children and young adults [30,62]. The burden of disability (YLD) was smaller than the burden of mortality (YLL), but it was not similar in all the geographic regions [19]. Since there are different ways to arrive at YLD, results from different studies cannot be readily compared. We used different disability weights for multiple health outcomes post-treatment, as opposed to the use of a single disability weight to estimate YLD for snakebites. The victims incurred significant out-of-pocket expenditure accessing the treatment. When healthcare and productivity costs were combined, the overall mean household-level loss was unexpectedly large for the study area. Our findings indicate that the average out-of-pocket expenditure in this study was nearly twice that reported recently from different districts in India [9]. The range of out-of-pocket expenditure in our study varied dramatically, spanning from USD 0 to USD 8477.44 across the district, reflecting substantial variation in treatment costs. This large variance suggests that our study probably included a large range of snakebite incidents, highlighting the importance of comprehensive cost assessments to fully understand and address the financial implications for the affected populations [63]. While there are some monetary compensation schemes [64] for snakebite-related deaths or disabilities in some parts of India, compensation schemes are often fraught with problems such as ambiguous eligibility criteria, inadequate compensation and delayed disbursement. This leads to incompatible policies and unmet needs in the real world. Notably, the financial burden borne by snakebite victims varies considerably across geographic regions. This variation is also consistent at different spatial scales [1,9] (Table 5). This results in localized disparities that are insufficiently addressed by the current institutional arrangements and compensation policies throughout India. Therefore, we advocate the standardization and stratification of the sampling approach to capture the true heterogeneity in the burden of snakebites so that healthcare accessibility and compensation mechanisms can be streamlined. As a starting point, the primary sampling unit should focus on the village level by involving the community members and health workers. We reiterate the importance of financial risk protection programs for snakebites and awareness campaigns directed at high-risk groups in rural areas.

### 3.5. Limitations

Several factors reduce the evidence value of survey studies on burden on disease, among them the prominent ones are: (i) Detailed clinical outcomes could not be verified due to incomplete records and limited disclosure by victims; future work should integrate hospital data for better validation; (ii) the verification of fatalities caused by snake envenoming; (iii) Snake species identification was primarily based on respondent recall and aided by photographs of common regional species; which may have introduced misclassification bias; (iv) the possible exaggeration of snake envenoming by inaccurate description of the incident and the outcomes. These are inherent problems associated with retrospective surveys related to snakebites. We have reduced the inaccuracies by cross-checking the reports with other independent members of the community. The identity of the species was verified by showing pictures of other venomous and non-venomous snake species. In several cases the snake that caused the bite was killed and brought to the hospital. To reduce the ambiguity in identification, we have assigned them as unidentified snake species. A selection bias is introduced by community-based snowball sampling, although it is useful for accessing a difficult-to-access population. The method is cost-effective, but the selection bias limits generalization to a broader scale [65,66]. It might have underrepresented victims who completely recovered or did not share their experience in the community. Further, there could be a recall bias associated with the retrospective self-reporting approach used in the study. We made an assumption that the demographic pattern for the study period was the same as in 2011, because the population census 2011 data was used. This assumption might have made the demographic classes not completely representative of the population. The financial burden could not be monetized separately for costs on antivenom, transport, or critical care charges. This might have underestimated the costs of treatment. By incorporating community-based sampling along with blinded stratified random samples and using simulations to arrive at selection probabilities, some of these biases could be addressed in future studies. Hybrid sampling designs are promising and might pave the way for accurate estimation of the burden of snakebites [27]. Such surveys need not be restricted to using only trained health workers. Including untrained community members such as village leaders, teachers, and victims can create a cascade of referrals, as demonstrated in this study. This could arrive at an accurate estimate of the economic burden of snakebites.

## 4. Conclusions

Snakebites cause a significant economic burden to the rural population in tropical countries, and they could also be an important factor that contributes to economic distress in the rural population linked to agricultural production. The high DALYs documented in this study underscore this impact. The biases of the sampling approach used in this study could be addressed by parametrizing inclusion probability and calibrating the weights for non-probability samples [67,68]. We highlight critical gaps in healthcare access, financial protection, and public awareness for snakebites at the district level. We further emphasize that a bottom-up approach of starting at the village level to document the burden and generating consensus on the approaches used to estimate the burden of snakebites is necessary before it can be scaled up.

## 5. Material and Methods

### 5.1. Study Area and Sampling

**Figure 1:**
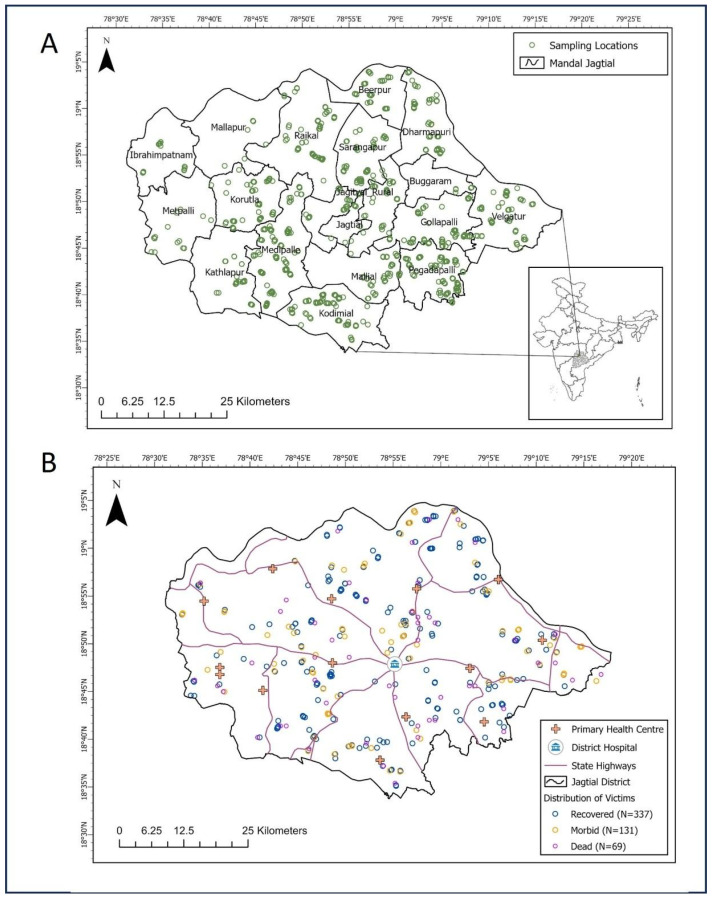
(**A**). Study area showing the mandals in Jagtial District and the sampling locations in the study. (**B**). Distribution of snakebite victims based on the outcome of the snakebite in Jagtial, India, from 2010 to 2020 documented in this study.

Between 2001 and 2014, Telangana State was among the top eight provinces with high snakebite fatalities [1]. Within Telangana, we selected Jagtial, a rural district, for the survey (Figure 1A). The district has 18 mandals (administrative units), each having about 20 to 30 villages. It had a population of 985,417 [69], with 484,079 males and 501,338 females, inhabiting 327 villages. Among them, 26 were urban, and we excluded them from the survey, as they experience low levels of snakebites. The sampling strategy employed was based on the principle where an initial set of known snakebite victims was recruited through the village head and subsequent victims were included through referrals [30,70,71,72]. The survey was terminated when there were no more new members included in the survey. We verified the cases of envenoming with available medical records, death certificates and first-hand witnesses of the incident from the community members. In a majority of the cases, the cause of death or morbidity could be clearly attributed to snakebites.

**Figure 2:**
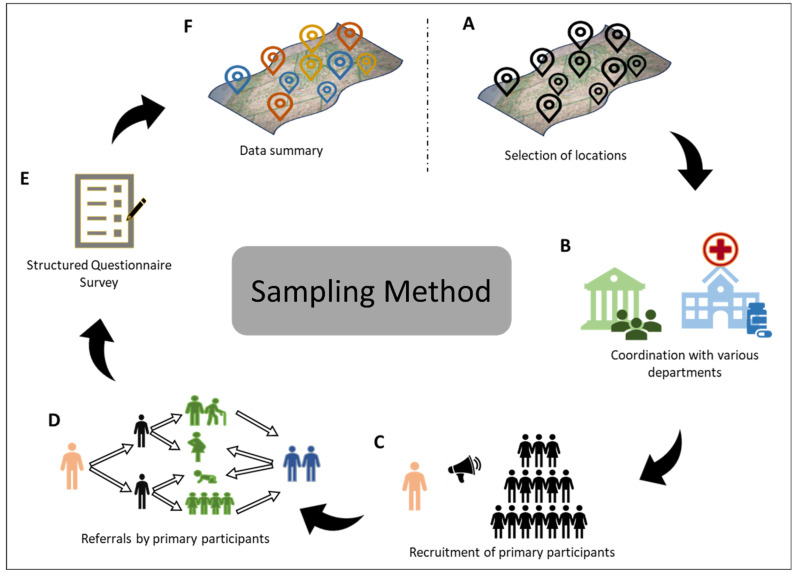
Graphical representation of methodology based on modified snowball sampling: (**A**) Out of the 301 rural villages, 205 were selected using random sampling across rural mandals for sampling in the study area (Jagtial district) as indicated by black locations. (**B**) The survey was initiated by involving the district public health machinery, which includes: District Magistrate and District Medical Officer, Accredited Social Health Activist (ASHA) workers, and village administrative officer, to elicit trust among the people and access to records. (**C**) A public message was broadcast by the village head (pink) one day before the actual survey, inviting all victims from 2010 to 2020 to participate. (**D**) The village head (pink) referred to an initial set of victims (black). They in turn referred to other victims (green) who were later recruited in the survey. This referral process was repeated until new victims (blue) were referred back to those who were already recruited in the survey. (**E**) A structured questionnaire was administered (in-person or via phone) in the local language—Telugu; the same was provided in Hindi and English after verbal consent. (**F**) Survey data were digitized, categorized, and mapped by outcome (recovered, morbid, deceased) for further analyses.

In cases where it was not clear, it was not considered a snakebite incident, and sampling was terminated. This sampling method is particularly useful in capturing rare entities in the sample from a population [73]. Non-probabilistic sampling methods select samples non-randomly (see Supplementary Table S1.4 for comparison), and it relies on existing study participants to recruit future participants through their acquaintances or knowledge [70]. It is employed in situations where participants might be reluctant to reveal their identity or their condition. A total of 541 participants were recruited for the survey for an 11-year investigation period from 2010 to 2020 (Figure 1). We randomly selected 205 of 301 rural villages for the survey. A step-by-step detail of the sampling used is described in Figure 2.

### 5.2. Data Collection and Management

Interviews were conducted involving victims or their relatives, accompanied by Accredited Social Health Activist (ASHA) workers and/or forest department staff. Most of the interviews were conducted in person; some were conducted by telephone with the consent of the village head. The questionnaire captured demographics, socioeconomic information, treatment charges, complications, and snake activity (see Supplementary S3). Although hospital reports and medical records were reviewed where available, some of the reporting of snake envenomation was based on self-reports provided by victims or their kin, without systematic physician verification. To enhance data reliability, self-reported information was cross-verified, where possible, through first-hand witnesses, ASHA workers, and available death certificates and medical records. The identification of snake species responsible for envenoming was based on victim or witness recall using vernacular names and observable characteristics. Variables such as education, bite site, delay in treatment, and effects after treatment were categorized (see Supplementary Table S1.5). We analyzed victims by their income, education, age, gender, and awareness. Individual costs (e.g., transport, ICU, ventilator) were not broken down, and the overall treatment cost was utilized to estimate the economic burden. This has been recognized as a limitation. Data on snakebite were also collected from the district hospital registers for six years (2015 to 2020), as the same data from 2010 to 2020 was not available. It had the number of patients, the victim’s address and date of admission. Treatment costs were not recorded in the registers. We performed quality checks on the dataset and cleaned the data for possible data entry errors. The number of victims recorded during the survey was segregated based on age, gender, type of house, occupation, educational qualification, outcome of the snakebite, type of the hospital visited to access treatment, and time required to access treatment.

### 5.3. Data Analysis

Chi-square test with a *p*-value < 0.05 was used to test for statistical associations. The differences in time taken to access treatment, number of hospitals visited, and time required to visit the hospital were tested using the Kruskal–Wallis non-parametric test followed by the Mann–Whitney U test with a *p*-value < 0.05. R program version 4.3.0 and Microsoft™ Excel version 2021 were used. The spread of snakebite incidents was mapped using ArcGIS pro software version 3.3.1. The age-adjusted population of the district was calculated based on the proportion of the age-adjusted population distribution of the district [69]. The epidemiology measure was calculated using the formula given in Supplementary S2.2 (S2.2.1–S2.2.3) using the computed age-adjusted population in Jagtial District and the WHO standard population for the villages where the questionnaire survey was carried out. Since the population data and life expectancy estimates were based on the 2011 Census, we made the assumption that this dataset would be representative of the study period. The information about the standard population was obtained from the WHO database [74].

The disability-adjusted life years (DALYs) were calculated using the formula mentioned in Supplementary S2.3 (S2.3.1–S2.3.4) using the age-adjusted Jagtial population for the selected villages. The years of life lost (YLL) were calculated using the life-expectancy data for each age extracted from GBD (Global Burden of Disease) 2021 [75]. We calculated the years lived with disability (YLD) by using the disability weights estimated in the GBD 2021 [76]. We assumed that snakebite envenoming and toxicity induced by other animals were comparable; therefore, a disability weight of 0.163 (ICD 10) was used [77]. We used disability weights for the health complications reported by the victims that led to long-term morbidity. We calculated separate YLD estimates for the recovered (*n* = 337) and morbid (*n* = 131) groups of the snakebite victims to understand the burden due to long-term morbidity. The duration of the recovery for victims was taken as 0.3 years as per GBD 2013 study [78,79], and life expectancy was taken as the duration of the disease for the morbid victims. We calculated YLL and YLD without age-weighting or discounting, setting both factors to zero [30,80]. We estimated the economic costs of earning opportunity cost and mortality cost. The earning opportunity cost was calculated using the earnings recorded from the minimum wages for each victim and the mortality cost was estimated using the per capita Gross Domestic Product (GDP) for each year. We used the minimum wage data from different sources [81,82,83] to measure the earning opportunity cost. It was recorded based on the specific occupation of the participant for each year. The mortality cost was calculated based on the GDP of India for each specific year [84]. The conversion of currency from Indian Rupees (INR) to United States Dollars (USD) was done utilizing the mean exchange rate of 82.572 INR/USD for 2023 [85]. Life expectancy measure for each year was used [75]. We reported the 95% confidence limits (CI) as CI: lower bound, upper bound. Monetary valuation was done using inflation adjustment for the study period [86].

## Figures and Tables

**Table 1:** The demographic parameters of the participants of the survey (N = 541) in Jagtial, India, from 2010 to 2020.

**Demographic Parameter**	**Frequency (%)**
**Gender**	
Male	314 (58.04%)
Female	227 (41.96%)
**Age Category (Years)**	
0–14	24 (4.44%)
15–59	477 (88.17%)
Above 60	40 (7.39%)
**House Type**	
Concrete House	178 (32.09%)
Traditional House	352 (65.06%)
Not Available	11 (2.03%)
**Occupation**	
Agriculture Sector	251 (46.40%)
Employed	191 (35.30%)
Unemployed	53 (9.80%)
Not in labour force	46 (8.50%)
**Education**	
≥10	142 (26.25%)
12	31 (5.75%)
Graduation	16 (2.96%%)
Post Graduation	2 (0.37%)
None	350 (64.70%)

**Table 2:** Estimated annual age-adjusted mortality rate caused by snakebites in Jagtial, India from 2010 to 2020.

Age Category (Years)	Jagtial Population *	Standard Population **	Observed Mortality	Expected Mortality	Observed Mortality Rate (per 100,000)
**0–6**	55,369	112,387	9	18.27	16.25
**7–9**	29,982	52,107	2	3.48	6.67
**10–14**	63,768	86,148	4	5.40	6.27
**15–19**	61,980	85,020	3	4.12	4.84
**20–24**	58,430	82,724	0	0.00	0.00
**25–29**	53,243	79,863	3	4.50	5.63
**30–34**	46,468	76,804	5	8.26	10.76
**35–39**	48,471	72,500	4	5.98	8.25
**40–44**	41,382	66,997	6	9.71	14.50
**45–49**	35,044	61,539	5	8.78	14.27
**50–54**	28,026	55,153	9	17.71	32.11
**55–59**	22,940	47,174	7	14.39	30.51
**60–64**	28,817	38,798	9	12.12	31.23
**65–69**	20,213	31,091	1	1.54	4.95
**70–74**	16,128	23,559	2	2.92	12.40
**75–79**	6905	16,521	0	0	0
**80+**	8346	11,615	0	0	0
**Not Stated**	14,387	0	0	0	0
**Total**	639,899	1,000,000	69	117.19	11.72
*Crude Mortality Rate* *(per 100,000)*			10.78	*Age-adjusted Mortality Rate*	11.72

* Age-specific population was calculated using age-adjusted proportions for the population of Karimnagar District, Census 2011, due to unavailability of the Jagtial District age- specific population data. ** WHO standard population (19).

**Table 3:** Estimated annual age-adjusted morbidity rate for snakebite in Jagtial, India, from 2010 to 2020.

Age Category (Years)	Jagtial Population *	Standard Population **	Observed Morbidity	Expected Morbidity	Observed Morbidity Rate (per 100,000)
**0–6**	55,369	112,387	0	0	0
**7–9**	29,982	52,107	1	1.74	3.34
**10–14**	63,768	86,148	1	1.35	1.57
**15–19**	61,980	85,020	1	1.37	1.61
**20–24**	58,430	82,724	4	6.85	6.85
**25–29**	53,243	79,863	2	3.76	3.76
**30–34**	46,468	76,804	10	16.53	21.52
**35–39**	48,471	72,500	8	11.97	16.50
**40–44**	41,382	66,997	21	34	50.75
**45–49**	35,044	61,539	18	31.61	51.36
**50–54**	28,026	55,153	21	41.33	74.93
**55–59**	22,940	47,174	26	53.47	113.34
**60–64**	28,817	38,798	13	17.50	45.11
**65–69**	20,213	31,091	1	1.54	4.95
**70–74**	16,128	23,559	3	4.48	18.60
**75–79**	6905	16,521	1	2.39	14.48
**80+**	8346	11,615	0	0	0.00
**Not Stated**	14,387	0	0	0	0.00
**Total**	639,899	1,000,000	131	227.83	20.47
*Crude Morbidity Rate* *(per 100,000)*			20.47	*Age-adjusted Morbidity Rate*	22.8

* Age-specific population was calculated using age-adjusted proportions for the population of Karimnagar District, Census 2011, due to unavailability of the Jagtial District age- specific population data. ** WHO standard population (19).

**Table 4:** Overall burden of disease due to snakebite in Jagtial, India, from 2010 to 2020.

	*Female*		*Male*		*Total*	
Age	Population	DALYs	Population	DALYs	Population	DALYs
**0–6 years**	27,194.52	376.22	28,139.33	199.27	55,369	575.49
**7–9 years**	14,805.50	81.78	15,160.49	63.10	29,982	144.89
**10–14 years**	31,673.22	112.58	32,065.22	122.44	63,768	235.02
**15–19 years**	30,963.35	59.74	30,993.34	121.81	61,980	181.55
**20–24 years**	29,045.35	0.20	29,359.00	39.41	58,430	39.61
**25–29 years**	27,401.77	51.20	25,845.99	110.05	53,243	161.25
**30–34 years**	24,284.85	131.45	22,199.22	159.62	46,468	291.06
**35–39 years**	25,744.40	50.27	22,755.44	145.06	48,471	195.34
**40–44 years**	20,668.76	217.17	20,698.20	130.26	41,382	347.43
**45–49 years**	17,616.71	62.51	17,417.70	162.37	35,044	224.88
**50–54 years**	14,148.53	172.44	13,871.92	195.45	28,026	367.89
**55–59 years**	13,106.72	102.44	9875.58	226.33	22,940	328.77
**60–64 years**	16,351.77	97.53	12,515.36	148.27	28,817	245.80
**65–69 years**	10,967.89	55.68	9264.07	9.61	20,213	65.29
**70–74 years**	7995.19	12.65	8125.10	45.37	16,128	58.01
**75–79 years**	3530.75	1.54	3374.12	0.10	6905	1.64
**80+ years**	4577.62	0.05	3778.08	0.15	8346	0.20
**Not stated**	7279.11	0.00	7105.84	0.00	14,387	0.00
**Total**	327,356.00	**1585.45**	312,544.00	**1878.67**	639,899	**3464.12**

**Table 5:** Comparative summary of three key studies estimating the epidemiological and economic burden of snakebite envenoming in India.

Parameter	Suraweera, W. et al. 2020 [1]	Menon, J. C. et al. 2025 [9]	This Study
Estimate Level	National	11 states, 25 districts	Single district
Mortality Rate per 100,000 population (min–max)	4.5 (0.7–8.9)	0.33 (0.05–1.18)	1.07
Morbidity Rate	Not specified	Not specified	2.07 per 100,000 population
Cost of Treatment in government and commercial hospitals *	Not specified	Government: INR 3900 Commercial: INR 27,400	INR 52,776.26 (USD 639.15)
Earning Opportunity Cost *	Not specified	Not specified	INR 26,528.60 (USD 321.27)
Mortality Cost *	Not specified	Not specified	INR 19,83,093.36 (USD 24,016.54)
DALY **	Not specified	Not specified	31.96 DALY per 100,000 population
Method used	Nationally representative survey with verbal autopsy	Community-based survey by ASHA health workers	Community-based survey by ASHA health workers, forest staff, and direct referral by victims
Biases	Misclassification errors from verbal autopsies leading to underreporting	Underreporting of snakebites	Recall bias in surveys leading to underestimation of the economic burden

* Mean annual per-person estimate. ** Annual estimate of DALY.

## Data Availability

The original contributions presented in this study are included in the article/Supplementary Material. Further inquiries can be directed to the corresponding author.

## Supplementary Materials

The following supporting information can be downloaded at: https://www.mdpi.com/article/10.3390/toxins18060250/s1, Supplementary Materials S1: Figure S1: Snakebite profile of the victims in Jagtial, Telangana. Figure A illustrates the different snake species identified by participants in the study, highlighting the diversity of snakes involved. Figure B details the specific body parts affected by snakebites, providing insight into common bite sites. Figure C maps the various locations where the incidents occurred, offering a geographical perspective on the data. Figure D captures the times of day when the snakebites occurred, reflecting any potential patterns related to timing. Figure E outlines the post-treatment h 14 ealth complications reported by victims, including common and severe symptoms. Figure F shows the time intervals between the snakebite and the participants’ arrival at the hospital, illustrating the delays experienced in seeking medical care; Table S1.1: Association of the time of snakebite with snake species using R software. Pearson’s Chisquared test; X-squared = 33.615, df = 10, *p*-value = 0.0002146; Table S1.2: Range of treatment cost paid by the snakebite victims based on the treatment they accessed and the hospitals they visited; Table S1.3: The annual mean earning opportunity cost and mortality cost incurred due to snakebite in Jagtial; Table S1.4: A comparison between the simple random sampling, and snowball sampling [70,71,72,87,88]; Table S1.5: The table categorizes variables reported by snakebite victims, including 46 education level, incident location, time to reach the hospital, and post-treatment health complications. Education level was grouped into five levels, location of the incident into nine specific categories, time into four intervals, and complications into six distinct health issues; Supplementary Materials S2: Definitions and Formulae; Supplementary Materials S3: Questionnaire for the survey.
