# Supplementary material for: Burden of Mortality and Morbidity Caused by Snakebites Contributes to Economic Loss in a Rural Population in India
Source: Toxins (Basel). 2026 May 29;18(6):250. doi: 10.3390/toxins18060250 (PMC13307855; doi:10.3390/toxins18060250)
Supplement: Supplementary file 1 [file toxins-18-00250-s001.zip › toxins-4311855-supplementary.pdf]

Supplementary Materials: Burden of Mortality and Morbidity Caused by Snakebites Contributes to Economic Loss in a Rural Population in India

Swapnil Kiran, Siripuram Srinivas and Karthikeyan Vasudevan

Supplementary S1

Figures and Tables

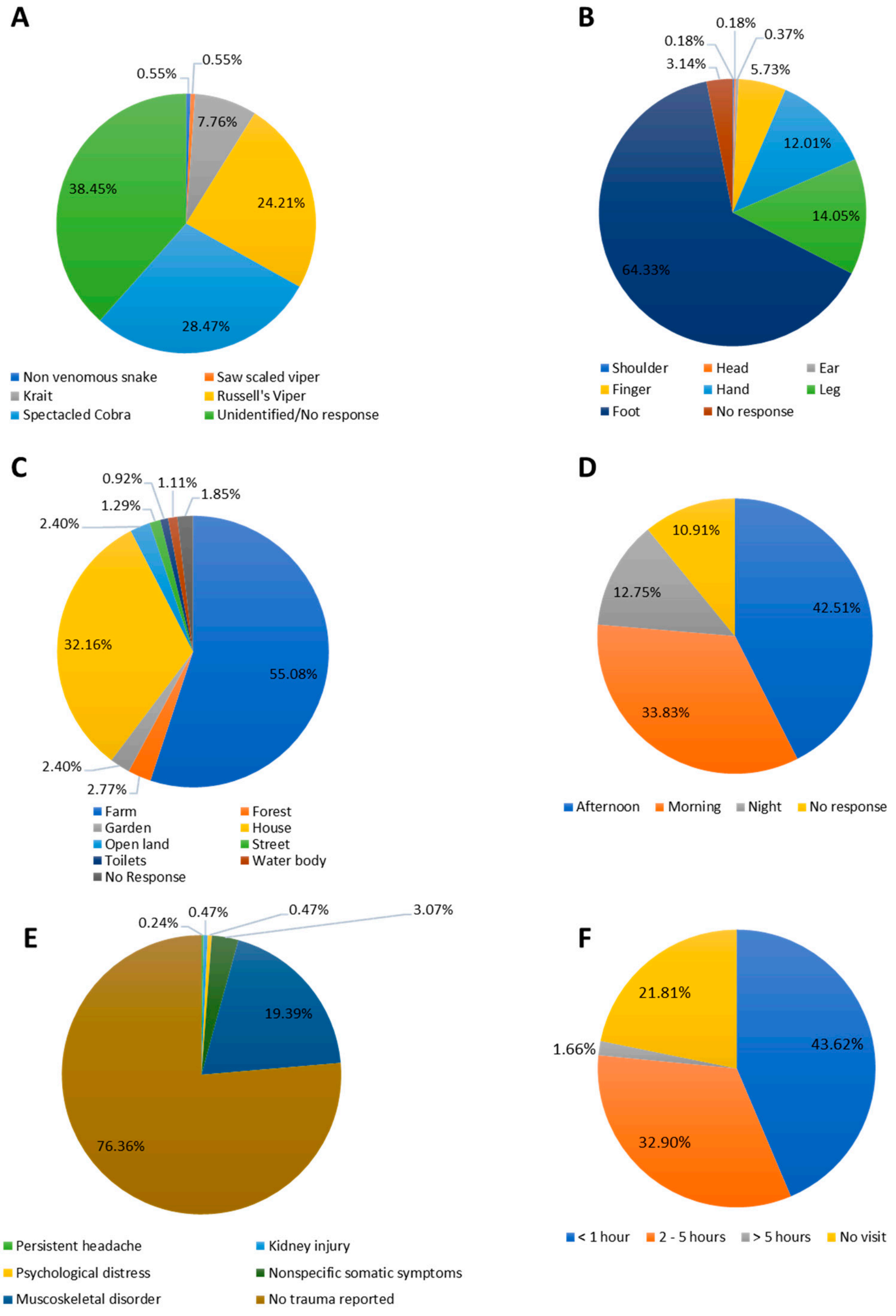

Figure S1: Snakebite profile of the victims in Jagtial, Telangana. Figure A illustrates the different snake species identified by participants in the study, highlighting the diversity of snakes involved. Figure B details the specific body parts affected by snakebites, providing insight into common bite sites. Figure C maps the various locations where the incidents occurred, offering a geographical perspective on the data. Figure D captures the times of day when the snakebites occurred, reflecting any potential

patterns related to timing. Figure E outlines the post-treatment health complications reported by victims, including common and severe symptoms. Figure F shows the time intervals between the snakebite and the participants' arrival at the hospital, illustrating the delays experienced in seeking medical care.

Table S1.1 : Association of the time of snakebite with snake species using R software. Pearson's Chi-squared test; X-squared = 33.615, df = 10, p-value = 0.0002146

	**Afternoon**	**Morning**	**Night**	**Unavailable**	**Grand Total**
**Krait**	13	10	16	3	42
**Non venomous snake**	1	2	0	0	3
**Russell's Viper**	63	42	15	11	131
**Saw scaled viper**	1	1	1	0	3
**Spectacled Cobra**	61	67	13	13	154
**Unidentified**	91	61	24	32	208
**Grand Total**	**230**	**183**	**69**	**59**	**541**

Table S1.2 : Range of treatment cost paid by the snakebite victims based on the treatment they accessed and the hospitals they visited.

Cost (INR)	Number of victims	Proportion
Faith Healer	**5**	**0.92%**
**0**	1	0.18%
**1 - 10000**	4	0.74%
Government Hospital	**109**	**20.15%**
**0**	81	14.97%
**1 - 10000**	21	3.88%
**10000 - 100000**	4	0.74%
**Unavailable**	3	0.55%
Health Centre	**5**	**0.92%**
**0**	4	0.74%
**1 - 10000**	1	0.18%
None	**113**	**20.89%**
**0**	52	9.61%
**1 - 10000**	46	8.50%
**10000 - 100000**	6	1.11%
**Unavailable**	9	1.66%
Private Hospital	**309**	**57.12%**
**0**	15	2.77%
**1 - 10000**	53	9.80%
**10000 - 100000**	173	31.98%
**100000 - 800000**	59	10.91%
**Unavailable**	9	1.66%
Grand Total	**541**	**100%**

Table S1.3 : The annual mean earning opportunity cost and mortality cost incurred due to snakebite in Jagtial.

Year	Mean earning opportunity cost INR (USD)	Mean mortality cost INR (USD)
**2010**	8,419.09 (101.96)	3,14,116.67 (3,804.15)
**2011**	8,535.64 (103.37)	3,24,864.39 (3,934.32)
**2012**	10,070.73 (121.96)	4,92,902.74 (5,969.37)
**2013**	16,217.13 (196.40)	8,01,470.50 (9,706.32)
**2014**	32,819.10 (397.46)	19,94,246.93 (24,151.61)
**2015**	47,159.73 (571.13)	59,14,930.13 (71,633.61)
**2016**	36,899.29 (446.87)	18,54,345.91 (22,457.32)
**2017**	29,863.61 (361.67)	30,67,216.12 (37,145.96)
**2018**	27,421.44 (332.09)	15,35,259.16 (27,421.44)
**2019**	43,250.51 (523.79)	35,04,340.48 (43,250.51)
**2020**	31,155.08 (377.31)	20,10,333.98 (24,346.44)
**Total**	26,528.30 (321.27)	19,83,093.36 (24,016.54)

Table S1.4 : A comparison between the simple random sampling, and snowball sampling.

Sampling approach	Advantages	Disadvantages
**Simple Random Sampling[87,88]**	**Representative Sample**: Simple random sampling ensures that each member of the population has an equal chance of being selected.	**Time and Cost:** Simple random sampling may require a large sample size to achieve accurate results, which can be time-consuming and costly
	**Unbiased Results:** Simple random sampling minimizes bias, as it eliminates any systematic selection bias that may occur in other sampling methods.	**Inefficiency:** In cases where the population is large and widely dispersed, simple random sampling may be inefficient as it requires reaching out to all individuals in the population
	**Statistical Inference**: Simple random sampling allows for statistical inference, where the findings from the sample can be generalized to the larger population	**Limited Precision for Small Populations:** Simple random sampling may not provide precise estimates for small populations since the sample size may not be large enough to capture the variation within the population
**Snowball Sampling based approach[70,71,72]**	**Access to Hidden Populations**: It is particularly useful for reaching	**Bias in Sample Selection**: It can lead to biased samples because the initial participants may refer

	populations that are difficult to identify or access.	others who are similar to themselves, resulting in a lack of diversity in the sample.
	**Rich Data Collection:** The method can lead to the collection of rich, qualitative data, as it often involves in-depth interviews and discussions that explore the social dynamics within networks.	**Difficulty in Estimating Population Size**: It can be challenging to estimate the total size of the population being studied.
	**Cost-Effective**: This sampling method can be more cost-effective than other sampling techniques, as it reduces the need for extensive initial screening or recruitment efforts.	**Ethical Considerations**: There may be ethical concerns regarding privacy and confidentiality, especially when participants are asked to refer others.

Table S1.5 : The table categorizes variables reported by snakebite victims, including education level, incident location, time to reach the hospital, and post-treatment health complications. Education level was grouped into five levels, location of the incident into nine specific categories, time into four intervals, and complications into six distinct health issues.

Variable	Inputs by participants	Grouped as
Education level	5th, 7th, 8th, 10th	$\leq 10^{th}$ standard
	11th, 12th, Diploma	12th standard
	B.Sc., B.Tech, Graduation	Graduation
	Post Graduation	Post Graduation
	No education, None, No	None
Location of the Incident	Field, agriculture field, near the field	Farm

	House, near the house, toilet inside house, kitchen	House
	Forest, near forest, inside forest	Forest
	Garden, house garden	Garden
	Open land	Open land
	Road side, street in the village, road outside forest	Street
	Near lake, near river, near canal	Water body
	Toilet outside the school	Toilet
	Do not remember	Unavailable
Time taken to reach hospital	30 minutes, 45 minutes, 50 minutes, less than 1 hour	Less than 1 hour
	Around 1.5 hours, 2 hours, 3 hours, 5 hours	1 to 5 hours
	6 hours, 8 hours, 12 hours, 24 hours	More than 5 hours
	No response, Not visited hospital	Not visited
Post treatment health complications	Pain & swelling occurs frequently, deformity of hand, inability to walk, numbness, amputation in toe, pulling sensation, heavy pain	Musco-skeletal disorder
	Generalised weakness, shivering, shortness of breath	Nonspecific somatic symptoms
	Haematuria	Kidney injury
	Fear of snakes, insomnia	Psychological distress
	Persistent headache	Persistent headache
	No response, no trauma experienced	No trauma reported

Supplementary S2

Definitions and Formulae

S2.1 Definitions

S2.1.1 Traditional House, dwelling typically constructed with non-durable, local materials such as mud, bamboo, thatch, or other natural substances. These houses are generally less permanent and more vulnerable to snakes and rodents as well as environmental conditions.

S2.1.2 Concrete House, dwelling constructed primarily using concrete, which is a composite material made from cement, water, and aggregates such as sand, gravel, or crushed stone. Concrete houses are known for their durability, strength, and resistance to environmental factors such as fire, termites, rodents, snakes and extreme weather conditions.

S2.2 Estimating epidemiological measures

S2.2.1 Cumulative Incidence is defined as the number of new cases with a condition in a population during a specified period of time. It is reported per 100,000 people in the article.

$$Cumulative\ Incidence = \frac{Number\ of\ \text{new}\ cases\ over\ a\ specified\ period\ of\ time}{Total\ Population}$$

S2.2.2 Mortality is related to the number of deaths caused by the event under study. It usually gets represented as a rate per 100,000 individuals, also called the death rate.

$$Mortality = \frac{Number\ of\ \ death\ cases\ over\ a\ specified\ period\ of\ time}{Total\ Population}$$

S2.2.3 Morbidity is the state of being symptomatic or unhealthy for a disease or condition. It usually gets represented as a rate per 100,000 individuals (morbidity rate).

$$Morbidity = \frac{Number\ of\ morbid\ cases\ over\ a\ specified\ period\ of\ time}{Total\ Population}$$

S2.3 Estimating burden of disease

S2.3.1 One **year of life lost** is represented by one YLL. The number of deaths multiplied by the worldwide standard life expectancy at the age of death gives the YLL.

*YLL (age, sex, external cause) = m (age, sex, external cause) * l*

where, m = Number of deaths, l = standard life expectancy.

S2.3.2 One **year lived with disability** represents the equivalent of one full year of healthy life lost due to disability or ill-health.

YLD (age, sex, external cause) = n(age, sex, external cause) l * {1-[(1-dws)*(1-dwp)]}*

where, n = number of cases, l = standard life expectancy, dw_s = short term disability weight, dw_P = permanent disability weight.

S2.3.3 Economic burden due to mortality: This burden encompasses the lost earnings and productivity of individuals who die prematurely, as well as the economic contributions they would have made over their remaining working years.

$$Mortality\ cost = S * \sum_{i=0}^{e} \frac{1}{(1+r)^n} * Y_i$$

where, S = total number of deaths, i = age of death, e = expected years of earning, r = discount rate (3%), and Y_i = GDP at year i^{th} year.

S2.3.4 Earning opportunity (EO) cost refers to the potential income lost by individuals who are unable to work due to being bitten by snakes. The financial burden due to immediate and extended loss of productivity.

$$EO\ cost = R * O$$

where, *R* = number of workdays lost due to recovery, *O* = average earnings per day.

Supplementary S3

S3. 1 Questionnaire for the survey

1. Name of Village
2. Name of the Mandal
3. Age
4. Gender
6. Education
7. Nature of Job
8. House Type
9. Latitude (House) in decimal degrees
10. Longitude (House) in decimal degrees
11. Outcome of the Bite
12. Type of Snake Bitten
13. Symptoms after the bite
14. Time of the incident
15. Bitten Body Part
16. Year
17. Location of the incident
20. Time required to Reach Hospital
21. Reason for delay
22. Actual Distance from Village to District Hospital (km)
23. Treatment Cost
24. Economic Status of victims
25. No. of Hospitals Visited
26. Hospital Type
27. Social Barrier (Y/N)
28. Awareness measure on First Aid
29. Post-treatment health Complications of Victims
30. Types of Snakes seen in the village
31. Python Sighting in the Village
